# Newer Oral Anticoagulants in the Treatment of Acute Portal Vein Thrombosis in Patients with and without Cirrhosis

**DOI:** 10.1155/2018/8432781

**Published:** 2018-06-05

**Authors:** P. Priyanka, J. T. Kupec, M. Krafft, N. A. Shah, G. J. Reynolds

**Affiliations:** ^1^Department of Medicine, West Virginia University Hospitals, Morgantown, WV, USA; ^2^Department of Medicine, Section of Digestive Diseases, West Virginia University Hospitals, Morgantown, WV, USA; ^3^Department of Medicine, Section of Hematology, West Virginia University Hospitals, Morgantown, WV, USA

## Abstract

**Background:**

Newer oral anticoagulants (NOACs) are being utilized increasingly for the treatment of venous thromboembolism (VTE). NOAC use is the standard of care for stroke prophylaxis in nonvalvular atrial fibrillation and treatment of acute VTE involving extremities and pulmonary embolism. In contrast, most guidelines in the literature support the treatment of acute portal vein thrombosis (PVT) with low molecular weight heparin (LMWH) and vitamin K antagonists (VKA). Literature evaluating NOAC use in the treatment of acute portal vein thrombosis is sparse. This review focuses on the safety and efficacy of the use of NOACs in the treatment of acute PVT in patients, with or without concomitant cirrhosis, based on the most recent data available in the current literature.

**Methods:**

A systematic review was conducted through a series of advanced searches in the following medical databases: PubMed, BioMed Central, Cochrane, and Google Scholar. Keywords utilized were as follows: NOAC, DOAC (direct oral anticoagulants), portal vein thrombosis, rivaroxaban, apixaban, dabigatran, and edoxaban. Articles related to newer anticoagulant use in patients with portal vein thrombosis were included.

**Results:**

The adverse events, including bleeding events (major and minor) and the failure of anticoagulation (propagation of thrombus or recurrence of PVT), are similar between the NOACs and traditional anticoagulants for the treatment of acute PVT, irrespective of the presence of cirrhosis.

**Conclusions:**

Newer oral anticoagulants are safe and efficacious alternatives to traditional anticoagulation with low molecular weight heparin and vitamin K antagonists in the treatment of acute portal vein thrombosis with or without cirrhosis.

## 1. Introduction

Portal vein thrombosis (PVT) is defined as thrombosis within the portal vein trunk and intrahepatic portal branches. The presence of PVT in cirrhosis varies with a reported incidence of 8.4% to 11.2% and prevalence nearing 26% [1]. The benefits of anticoagulation are seen in both cirrhotic and noncirrhotic patients. These benefits include a higher rate of recanalization (42-100%), a lower thrombus extension rate, a lower incidence of hepatic decompensation, and improved survival [2–7].

Historically, patients with liver dysfunction were advised to avoid newer oral anticoagulants due to the risk of excess bleeding and reduced effectiveness. However, increasing evidence suggests that oral anticoagulants are not only safe but also efficacious when compared to the traditional anticoagulation [8–12].

Although the exact duration of anticoagulation for acute portal vein thrombosis remains controversial, the American Association for the Study of Liver Disease (AASLD) recommends at least three months of anticoagulant use in the treatment of PVT, irrespective of the presence of cirrhosis [13]. Most trials of treatment of PVT have effectively used low molecular weight heparin and warfarin [5, 14, 15]. Far fewer studies have examined the safety and efficacy of newer oral anticoagulants (NOACs) in the treatment of this disease process.

NOACs are one of the landmark advances in the recent practice of medicine as they overcome numerous drawbacks of traditional anticoagulants. NOACs are currently approved for stroke prophylaxis in nonvalvular atrial fibrillation, venous thromboembolism (VTE) prophylaxis in orthopedic surgery patients, and the treatment of VTE involving the extremities and acute pulmonary embolism (PE) [16]. Despite this progress, anticoagulation of portal vein thrombosis with NOACs has remained controversial. Traditionally, low molecular weight heparin (LMWH) and vitamin K antagonists (VKAs) have been used mainly due to lower costs, more physician experience with these agents, and easy reversibility in case of severe bleeding [5, 6]. The role of NOACs for the PVT treatment is still undefined in the current literature.

The complications of PVT (gastrointestinal bleeding, including variceal, intestinal infarction and development of portal hypertension, portal cholangiopathy, and subsequent hepatic decompensation) can be quite serious [1]. Recanalization of the thrombus occurs more frequently with the aid of anticoagulation thereby reducing complications mentioned above. Additionally, the development of PVT is associated with higher morbidity and mortality after liver transplantation, complicating treatment strategies [17]. As supported by animal data, another proposed benefit of anticoagulation in PVT is the suggestion that fibrogenesis may be reduced by thrombin antagonism [17, 18].

Patients with cirrhosis have been excluded* ipso facto* in clinical trials of NOACs mostly due to concerns of severe bleeding in the absence of reversal agents. Off-label use of NOACs in VTE involving atypical sites like splanchnic vein thrombosis, cerebral venous thrombosis, and renal and ovarian vein thrombosis has been evaluated with excellent efficacy and safety profile [19]. Despite the ever-increasing evidence, NOACs have not yet found mainstream success as an alternative for the treatment of PVT [13].

The purpose of this review is to evaluate the existing literature on the use of NOACs for PVT in patients with and without cirrhosis.

## 2. Methods

A comprehensive, systematic search of the PubMed, Biomed Central, Cochrane Library, and Google Scholar was performed. Relevant English language articles were identified up to February 28, 2018. Manual searching for relevant publications from the reference section of extracted articles was also performed.

A total of 53 manuscripts were identified after searching the above databases. Duplicate studies were removed. Of these articles, 43 were discarded as they were not relevant to the topic and 10 were included (Table 1).

Of the selected 10 articles, four studies were original manuscripts and six were case reports. Only one study was prospective and three were retrospective. Six studies involved patients with cirrhosis, three articles included only noncirrhotic patients, and one study included patients both with and without cirrhosis. In all, a total of 119 patients, across all studies, were included.

Recanalization rates and thrombus recurrence rates served as efficacy endpoints. Bleeding events were utilized as the complication endpoint.

## 3. Drawbacks of Traditional Anticoagulants Use in PVT with Cirrhosis

Despite their acceptance as the standard of care for many thromboembolic events, there are some disadvantages of using LMWH and VKAs in the treatment of PVT.

For example, in patients with cirrhosis, the efficacy of unfractionated heparin (UFH) and LMWH may be significantly decreased (up to 40%) due to lower levels of antithrombin synthesis by the liver [24]. Additionally, LMWH has limited use in concurrent renal impairment.

Coagulopathy from liver disease frequently results in an elevated INR and thus utilizing the INR to guide dosing of VKAs is particularly challenging. Following the INR as a monitoring parameter was only studied in noncirrhotic population and is being used by extrapolation for VKA monitoring in patients with cirrhosis [8]. Elevated INR levels associated with cirrhosis can offer a false measure of the therapeutic efficacy. Conversely, when VKAs artificially elevate the INR, this can directly affect the model for end-stage liver disease (MELD) score, impacting transplantation eligibility. Protein C, a vitamin K dependent anticoagulant factor, is reduced in cirrhosis and may decrease the efficacy of VKAs [8].

VKAs have a narrow therapeutic window as well as numerous drug and dietary interactions and need frequent coagulation testing and dose adjustments. Despite their efficacy, VKAs are far from an easy means of anticoagulation.

## 4. Advantages of NOACs for VTE

There are benefits to considering NOACs for the treatment of thromboembolic events. These medications can be cost-effective and convenient. Unlike LMWH, they do not require daily subcutaneous injections, and since their pharmacokinetics and pharmacodynamics are predictable, they do not require routine dose monitoring like warfarin. In contrast to the strict dietary precautions VKA users have to follow, NOACs pharmacodynamics are not affected by dietary intake [25]. In addition, NOACs act faster than VKAs. Rivaroxaban's onset of action is 30 minutes, faster than the 36-72 hours for warfarin (which requires overlap with heparin therapy). Interactions of NOACs with other drugs are rare, with the exception of a few antibiotics and antifungals, which may be significant for patients with malignancy [26].

Systemic clearance upon discontinuation of NOACs is faster (5-9 hours in young adults and 11-13 hours in older adults) and more reliable when compared to warfarin (20-60 hours) [27]. Clinically this is significant as rivaroxaban can be stopped much closer to an elective surgery or invasive procedure than warfarin. All of these properties combine to increase patient compliance, reliability, and effectiveness.

## 5. Rivaroxaban Is the Most Studied NOAC for the Treatment of PVT

Rivaroxaban has been consistently used in the most studies examining the role of NOACs in the treatment of PVT either as a single agent or in combination with a second agent such as apixaban or dabigatran [8–12, 19, 21–23]. Similarly, the studies evaluating the pharmacodynamics and hepatotoxicity of NOACs in patients with cirrhosis were mainly performed with rivaroxaban [28, 29].

## 6. Summary of the Data Supporting Treatment of PVT with NOACs (Table 1)

There is little scientific evidence regarding the use of NOACs in PVT, and well-designed prospective studies are even fewer. Recently, a prospective study involving patients from the Mayo Clinic thrombophilia registry for initial treatment of acute venous thromboembolism of atypical location (VTE-AL) was published [19]. Outcomes, recurrence of PVT, and bleeding rates were similar for rivaroxaban and apixaban for patients with VTE in a typical location (VTE-TL) treated with NOACs compared to VTE-AL treated with enoxaparin in 623 patients without cirrhosis. In this study group, 29 had PVT and 63 patients had VTE-AL. Another multicenter, retrospective European study with 94 patients from 17 centers looked at NOAC use for splanchnic vein thrombosis, cardiac arrhythmias, peripheral deep vein thrombosis (DVT), and PE [19]. They concluded that NOACs were an effective and safe alternative to LMWH and VKAs, including patients with PVT with and without underlying cirrhosis.

Smaller studies aimed at evaluating the risk of bleeding in patients with PVT treated with NOACs show near equal complications to those treated with LMWH and VKAs [8, 9]. It must be noted, however, that patients with decompensated cirrhosis were mostly excluded from these studies with only a few Child Pugh Turcotte (CPT) class C patients included in just one study [9].

## 7. Special Considerations of NOACs in PVT

PVT can be associated with thrombosis of the mesenteric vein. Mesenteric vein thrombosis may cause impaired drug absorption due to reduced mesenteric perfusion resulting from severe venous congestion [19]. This could interfere with the efficacy of NOACs. It is important to be aware of this possibility while initiating treatment with NOACs in patients with PVT.

Given the progressive nature of cirrhosis, hepatic function may change over time. During long-term treatment with NOACs, this progression could potentially be missed as routine coagulation testing is not recommended with NOACs. This emphasizes the need for detailed testing of the liver function prior to initiation of treatment and also supports periodic monitoring of coagulation parameters while on NOACs [30].

A recent study suggests reduced in vitro anticoagulant potency of rivaroxaban in patients with CPT B and C cirrhosis and increased anticoagulant effect of dabigatran proportional to the severity of liver disease [31]. This may result in overanticoagulation or even undertreatment. Measurement of factor X levels for rivaroxaban and factor II levels in dabigatran may be necessary in cirrhosis patients to ensure the therapeutic efficacy. This highlights the need for agent-specific dose adjustments in the presence of liver cirrhosis.

## 8. Local Anticoagulant Action of Factor Xa Inhibitors in PVT

It has been postulated that reduced flow rates in the portal vein in PVT or cirrhosis may be associated with severe protein C deficiency contributing to a locally prothrombotic milieu [32]. Due to the very high bioavailability of rivaroxaban and other factor Xa inhibitors, local anticoagulant action of these agents compared to systemic anticoagulants has been proposed, as the metabolites of rivaroxaban have no anticoagulant activity [11]. This could support the use of even lower doses (2.5 mg once or twice daily) of rivaroxaban in PVT.

## 9. Agent-Specific Considerations in NOACs

### 9.1. Rivaroxaban, Apixaban, and Edoxaban

Rivaroxaban, apixaban, and edoxaban are direct factor Xa inhibitors with half-lives of 5-9 hours, 12 hours, and 10-15 hours, respectively. Rivaroxaban and apixaban are metabolised in the liver (67%) and kidneys (33%) and are extensively bound to plasma proteins. Edoxaban is metabolised hepatically and renally equally. Each of these agents is well absorbed in gastrointestinal (GI) tract and has high bioavailability. Rivaroxaban absorption occurs only in the stomach making it the better choice for patients with gastric tubes. Apixaban is absorbed through the entire length of GI tract, predominantly in the distal small bowel and ascending colon. As a result, apixaban administration may not be suitable for patients with a proximal colectomy or a distal small bowel resection. Edoxaban is absorbed mainly in the small intestine; therefore it can be given effectively to patients with previous colectomy [33–37].

## 10. Dabigatran

Unlike the factor Xa inhibitors, dabigatran has unique pharmacodynamics. Dabigatran has poor absorption in the gastrointestinal tract, limited hepatic metabolism, minimal binding to plasma proteins, and a longer half-life (12-14 hours) and is eliminated almost entirely through the kidneys (80%) [38]. Additionally, dabigatran has a different mechanism of action; it is a direct thrombin inhibitor [39, 40]. It is known to cause significant dyspepsia due to high concentrations in the colon secondary to poor GI absorption and tartaric acid content of the capsule [41]. It becomes clinically significant as simultaneous administration of pantoprazole reduces dabigatran's effective therapeutic area under curve (AUC) by 22%. At this time, dose modification of dabigatran in CPT A and B cirrhosis patients is not indicated due to limited data available on its use in these patients.

## 11. Efficacy of NOACs in Acute PVT

Anticoagulant efficacy can be defined as both the rate of recanalization of the thrombus in the portal vein and reduction in recurrent thrombi formation. NOACs appear to perform as well as traditional anticoagulants in achieving recanalization and have similar thrombus recurrence rates [9, 19]. In the aforementioned Mayo oncology study, recurrence of VTE-AL was 7.3% in patients with underlying malignancy treated with rivaroxaban [19]. This was not different from recurrence rates noted in VTE-TL. NOAC use in splanchnic vein thrombosis, a study by the Vascular Liver Disease Group (VALDIG) consortium that included patients with and without cirrhosis, showed thrombotic events in two of 58 treated patients (3.4 %), including a case of progression of PVT [10]. No anticoagulation failure was reported in the cirrhosis group in this study. In another study, only one failure of anticoagulation in both the NOAC and the traditional anticoagulant groups was observed in 27 patients with cirrhosis treated for various indications [9]. Additionally, no progression of PVT was noted. Complete recanalization of PVT has been noted in case reports; one report describes partial recanalization [11, 12, 20–22].

## 12. Monitoring of NOACs

In 2014 guidelines from the Australasian Society of Thrombosis and Hemostasis, prothrombin time prolongation was thought to be most sensitive test for rivaroxaban and edoxaban, while activated partial thromboplastin time (aPTT) and thrombin time (TT) were markers of dabigatran [42]. HEMOCLOT® direct thrombin inhibitors assay (HYPHEN BioMed, France, CK002K) is the recommended confirmatory test for drug levels of dabigatran. Drug-specific anti-factor Xa assays are recommended for apixaban, edoxaban, and rivaroxaban for confirmation. The assays for these agents are not currently commercially available and they are of little use in emergent situations. Furthermore, it is important to note that a normal aPTT with dabigatran or normal PT with apixaban, edoxaban, or rivaroxaban does not exclude the presence of active drug [43].

## 13. Indications and Duration of NOAC Treatment in PVT

AASLD guidelines recommend at least three months of anticoagulation with traditional anticoagulants for all patients with acute PVT irrespective of presence of symptoms. Long-term treatment should be considered in patients with permanent risk factors for thrombosis and concomitant mesenteric vein thrombosis, due to the risk of mesenteric infarction [40].

The American College of Chest Physicians recommends a minimum of three months of anticoagulation for symptomatic patients only and no anticoagulation for asymptomatic patients [16]. Indefinite anticoagulation should be considered in patients with permanent risk factors such as cirrhosis, malignancy, or autoimmune disorders.

The duration of anticoagulation with NOACs has not been addressed in any guideline as NOACs are still not the standard of care in PVT treatment. In the studies discussed in this review, the duration of treatment varies from 5 to 13 months in duration and longer in some cases.

## 14. Adverse Events Noted in the Studies Involving NOACs in PVT

### 14.1. Bleeding

As with any form of anticoagulation, the risk of bleeding should be balanced with the potential benefit of treatment. A few studies have evaluated the overall risk of bleeding when utilizing newer oral anticoagulants. Major bleeding was defined as overt bleeding with a drop in the hemoglobin greater than or equal to 2 g/dl, transfusion of two or more units of packed red blood cells, or intracranial, intraspinal, intraocular, retroperitoneal, pericardial, or fatal bleeding.

Any bleeding events not meeting the above criteria, but requiring medical attention, were categorized as nonmajor bleeding.

### 14.2. Major Bleeding

In the available studies, the rate of major bleeding with NOACs was not higher than with traditional anticoagulants. In patients treated with apixaban and rivaroxaban from the Mayo Thrombophilia Registry, two patients out of 36 treated with NOACs (5.55%) with underlying malignancy had a major bleeding event [19]. In the VALDIG study, major bleeding requiring discontinuation of NOAC was seen in two of 258 (0.71%) patients without cirrhosis and one of 36 (2.7%) patients with cirrhosis [10]. These included bleeding after variceal band ligation and after hysterectomy in the noncirrhosis cohort and lower GI bleeding in the cirrhosis group. These rates are not higher than major bleeding rates with traditional anticoagulants. No excessive major bleeding rates were noted in two other studies specifically designed to investigate the risk of bleeding in cirrhotic patients with NOACs when compared to traditional anticoagulants [8, 9]. Due to differences in the dosing regimens and indications for anticoagulation, investigators could not ascertain if the risk of bleeding was uniform in their cohort. Some data actually suggests that the risk of major bleeding was higher with traditional anticoagulant use (28%) than NOACs (4%) in patients with cirrhosis [9]. This reduced risk was specifically noted for central nervous system (CNS) bleeds, as no CNS bleeding was noted with NOAC therapy compared to three (17%) cases for traditional anticoagulants. Only one case report recorded hematemesis and melena but no other major bleeding events were noted in any case reports [11, 12, 20–23].

### 14.3. Nonmajor Bleeding

Nonmajor bleeding rates were actually lower in patients with VTE-TL treated with NOAC (8.7%) in the Mayo study than the nonmajor bleeding rate in patients with VTE-AL treated with enoxaparin (15.6%) [19]. The VALDIG study showed similar nonmajor bleeding rates in patients with cirrhosis (11.1%) and without cirrhosis (12%). These bleeding events included epistaxis, gingival bleeding, GI bleeding in the noncirrhotic group and bleeding from portal hypertensive gastropathy, lower GI bleeding, epistaxis, and bleeding after band ligation in the cirrhosis cohort. In another study, minor bleeding events included vaginal bleeding and GI bleeding [8].

A recent meta-analysis from 43 randomized controlled trials has concluded that there is an increased risk of gastrointestinal bleeding with NOACs use with an incidence of 1.4% [44]. Furthermore, this risk was found to be more for rivaroxaban and dabigatran and less for edoxaban and apixaban. Concurrent antiplatelet therapy can further increase the bleeding risk by five times. Currently, although there is no evidence in literature to suggest that anticoagulation increases the risk of variceal bleeding, studies recommend screening for varices and initiating or optimizing beta blocker therapy or perform endoscopic variceal ligation (EVL) before starting anticoagulation [45, 46].

## 15. Other Side Effects

While much of the risk-benefit discussion with the patients centers on the risk of bleeding, additional adverse effects (AEs) of NOAC treatment should be considered. As these drugs are relatively new, the amount of data available is limited. Some of these AEs were elucidated by VALDIG investigators. In patients without cirrhosis, leukopenia (n=1) was a rare event but was not severe enough to discontinue treatment. However, dizziness (n=1) did require discontinuation of the medication [10].

## 16. Other Limitations of NOACs

### 16.1. Higher Cost

New medications often face challenges with respect to insurance coverage and overall costs to the patients and NOACs are no exception. Higher costs are a concern with NOACs, but it has been shown that these costs can be offset by reduced costs of monitoring, less healthcare provider time, and an increased convenience to the patient. As such, some data supports the idea that rivaroxaban can be more cost-effective than warfarin in the prevention of recurrent VTE [47].

### 16.2. Hepatotoxicity

Interestingly, no cases of hepatotoxicity were noted in the studies focusing on NOAC use in PVT, possibly due to smaller size of these studies. Liver toxicity of NOACs, particularly rivaroxaban, more so than dabigatran or apixaban, has been reported in the literature. The incidence of hepatotoxicity in patients with chronic advanced liver disease is estimated to be 0.1-1% and appears to be idiosyncratic in nature [28, 29]. Liver dysfunction is usually reversible after stopping the treatment. Though infrequent, patients should be made aware of the possible symptoms of liver dysfunction.

### 16.3. Cautious Use in Liver Dysfunction

Moderate or severe hepatic impairment can reduce NOAC clearance and augment their pharmacodynamic effects to varying degrees. Significantly increased exposure was noted in CPT B cirrhosis patients compared to CPT A patients for rivaroxaban [48]. In patients with CPT B cirrhosis, the area under the plasma concentration or AUC of rivaroxaban and apixaban is increased (2.27 times and 1.09 times, respectively) and decreased (4.8% and 5.6%) for edoxaban and dabigatran, respectively [30]. In comparison, there was no difference in the drug exposure between patients with mild and moderate hepatic dysfunction after administration of a single 15 mg dose of edoxaban, indicating more consistent drug pharmacokinetics [49]. Currently, rivaroxaban and edoxaban are recommended to be avoided in patients with CPT B and C cirrhosis and apixaban in patients with CPT C cirrhosis [33–35]. There are risks of higher doses of NOACs, particularly in prolonged exposure in patients with liver dysfunction, but the data is limited with respect to recommending a dose adjustment in moderate to severe liver dysfunction with any of these drugs [11]. These factors should be considered when choosing a NOAC in patients with cirrhosis.

### 16.4. Cautious Use in Renal Impairment

Since all NOACs are excreted through kidneys to some degree, caution needs to be exercised in utilizing NOACs in the presence of renal impairment. The Food and Drug Administration (FDA) has approved a lower dose of dabigatran (75 mg bid), apixaban (5 mg bid or 2.5 mg bid), and rivaroxaban (15 mg od) for patients with renal insufficiency and creatinine clearance of 15-30 ml/min [33, 34, 38]. NOACs are not indicated at this time in end-stage renal disease (ESRD) and patients on hemodialysis.

### 16.5. Reversal

One of the notable advantages of the traditional anticoagulants is the availability of reversal agents. This is significant in the context of NOACs where a lack of a cost-effective, widely available reversal agent hampers their use with respect to major bleeding events. To reduce the risk of excessive bleeding during surgery, adequate reversal of NOACs is of paramount importance in patients awaiting liver transplantation.

A recent review supported the superiority of four-factor prothrombin complex concentrate (4F-PCC) over fresh frozen plasma (FFPs) as a reversal agent [50]. Determining the appropriate dose of 4F-PCC remains a challenge as excessive use may result in a higher risk of thrombosis. Idarucizumab, a monoclonal antibody for dabigatran reversal, used as a single intravenous dose has been shown to be effective. Though available in many countries, use of both PCC and idarucizumab remains limited as they may be cost prohibitive [51]. Andexanet alfa, a recombinant modified human factor Xa decoy protein, though not yet approved, has shown potential as a possible reversal of factor Xa inhibitors. Ciraparantag binds directly to factor Xa inhibitors, dabigatran, LMWH, and unfractionated heparin and is under evaluation for reversal of both direct thrombin inhibitors and factor Xa inhibitors [51].

In addition, dabigatran is amenable to neutralization with gastric lavage soon after ingestion and, due to its low protein binding and lipophilic nature, hemodialysis in severe cases.

## 17. Dosing of NOACs for PVT Patients

The presence of cirrhosis may affect the dosing of NOACs. Yet current data is unclear in determining the appropriate dose. In the VALDIG study, the median daily dose of all NOACs (rivaroxaban, apixaban, and dabigatran) in patients without cirrhosis was 25% higher than patients with cirrhosis [10]. In another study, involving 20 patients with VTE, including 12 patients with splanchnic vein thrombosis, 75% were treated with therapeutic dose of rivaroxaban (20 mg daily) and apixaban (5 mg twice a day), and 25% were treated with lower dose (rivaroxaban 10 mg daily and apixaban 5 mg daily) successfully without excessive bleeding episodes and recurrence rates [8]. Other studies were performed without dose modification [9]. Case reports support a standard dose of rivaroxaban, even in the presence of cirrhosis, without any effect on efficacy and bleeding events [11, 12]. At this time, there is no clear guidance in the literature about the effect of dose modification on the efficacy and possible benefit for cirrhosis patients.

## 18. Bridging NOACs with Traditional Anticoagulants before the Initiation of PVT Treatment

Traditionally, VKAs are bridged with unfractionated heparin (UFH) or LMWH for five days. For treatment of VTE in typical locations, NOACs do not routinely require heparin bridging. At this time, the data regarding bridging NOACs with UFH/LMWH is limited and is currently subject to the physician preference.

The available studies and case reports support several approaches to potentially utilizing another agent to bridge patients before starting NOAC, and some of them do not address bridging at all [8, 19]. NOAC was used as the initial anticoagulant in one-third of patients without cirrhosis while two-thirds were previously treated with traditional anticoagulants, without any mention of bridging [8]. Other studies did not bridge their patients with traditional anticoagulants [9]. There are case reports in this review where patients were bridged with heparin and some in which heparin was not used [11, 23].

## 19. Limitations

There are some limitations to the studies included in this review. First, it is significant heterogeneity which makes comparison between studies difficult. There are differences in baseline patient characteristics, the indications for anticoagulation, the anticoagulant agents used, the doses given, and the primary endpoints. It is difficult to standardize a review when such parameters do not align. The second major limitation is the quality of the studies available. None of the included studies were randomized controlled trials, and, barring one study, all were retrospective. The study sizes were small. These were not controlled studies as the anticoagulation prescribing (initiation, choice of the agents, discontinuation, and transitioning to and from traditional anticoagulants due to intolerance or adverse effects) was not under the investigators' control. Many studies did not have a control group. Additionally, patients with CPT C cirrhosis were largely excluded. Patients with mesenteric ischemia or renal impairment were also excluded from these studies limiting our insight into management of these subgroups with NOACs.

## 20. Conclusions and Future Directions

Although the current literature does not conclusively establish the role of NOACs in treatment of PVT in the presence or absence of cirrhosis, it extends the armamentarium of safe and efficacious options available for anticoagulation of these patients.

This review establishes that NOACs can be used effectively and safely without any risk of increased bleeding events in the treatment of PVT, even in patients with CPT class A and B cirrhosis. This review also presents the evidence that NOACs are already being increasingly used off label for this indication despite the lack of robust data on their safety and efficacy. This preliminary data may prompt better quality studies in the future comparing traditional and newer anticoagulants.

## Figures and Tables

**Table 1:** 

Author	Study design	Number of patients (n)	Duration	Agent	Response	Bleeding events
Janczak [19] (2018)	Prospective, non-cirrhotic, Atypical sites	Total N=36 PVT N= 16		Rivaroxaban Apixaban	Recurrence rate 7.3 % (n=2) (both had malignancy)	Minor N=1 (3.6%) Major N=2 (7.2%)

Qi [20] (2017)	Case report, cirrhotic, CPT not specified	SMV, splenic vein, N=1	11 weeks	Rivaroxaban	Recanalization	Melena and hematemesis

Nery [21] (2017)	Case report Non-cirrhotic	N=1	>6m	Rivaroxaban 20 mg daily	Complete recanalization L branch, partial recanalization right branch	None

De Gottardi [10] (2016)	Retrospective, Both cirrhotic and non-cirrhotic, Splanchnic	Total, N= 94 PVT N= 80 (Non cirrh- N=38 Cirrh- N=22)	Non-cirrhotic 13.1 m cirrhotic 9.6 m	Rivaroxaban Apixaban Dabigatran	Not studied	Cirrhosis: Minor, n=7, Major, n=2 Non-cirrh: Minor, n= 4 Major, n=1

Hum [9] (2016)	Retrospective, Cirrhotic, CPT A, B & C All indications	Total, N= 27 PVT N=4		Rivaroxaban 15 mg bid +/- 20 mg daily load, Apixaban 5mg bid +/- 10mg bid load, No bridging	Recurrence rate 4% (n=1)	Major N=1 (4%) Minor, N=7

Yang [12] (2016)	Case report, cirrhotic CPT A	N=1	6 m	Rivaroxaban 15 mg bid x 3 wks, then 20 mg/d	Complete recanalization	None

Intagliata [8] (2015)	Retrospective, cirrhotic, CPT A and B	Total N= 20 PVT, N=12	10.6 m	Apixaban Rivaroxaban	Not studied	Major, n=1

Martinez [22] (2014)	Case report, Cirrhotics, CPT A	N=1	6 m	Rivaroxaban 20 mg /d	Complete recanalization	None

Lenz [11] (2014)	Case report, Cirrhotic CPT A	N=1	5 m	Rivaroxaban 10 mg daily	Complete recanalization	None

Pannach [23] (2013)	Case report, Non-cirrhotic	N=1	>4 weeks	Rivaroxaban 20 mg daily	Complete recanalization	None

CPT: Child Pugh Turcotte; Cirrh: cirrhosis.
